# Total Flavonoids of *Rhizoma drynariae* Enhance Bone Marrow Mesenchymal Stem Cell-Mediated Tendon–Bone Healing by Promoting Tissue Regeneration, Angiogenesis, and Modulation of Cytokine Expression

**DOI:** 10.3390/biology14111593

**Published:** 2025-11-14

**Authors:** Gaoyuan Yang, Yu Wang, Xianyan Xie, Ziyan Li, Shuqi Qin, Weitong Zhang, Zixi Chenyuan, Peizhong Cao, Huiguo Wang, Lin Zhu

**Affiliations:** 1 School of Sport and Health, Guangzhou Sport University, Guangzhou 510500, China; 2 Research Center for Innovative Development of Sports and Healthcare Integration, Guangzhou Sport University, Guangzhou 510500, China; 3 Innovative Research Center for Sports Science in the Guangdong-Hong Kong-Macao Greater Bay Area, Guangzhou Sport University, Guangzhou 510500, China

**Keywords:** tendon–bone injury, total flavonoids of *Rhizoma drynariae*, bone marrow mesenchymal stem cells, tissue regeneration

## Abstract

(1) Objective: This study aimed to investigate the synergistic effect and underlying mechanisms of Total Flavonoids of *Rhizoma drynariae* (TFRD) in combination with Bone Marrow Mesenchymal Stem Cells (BMSCs) in the repair of tendon–bone injuries. (2) Methods: The effects of TFRD on the proliferation and migration of BMSCs were assessed using CCK-8 and scratch assays, and its potential to promote osteogenic and chondrogenic differentiation was evaluated. Concurrently, the pro-angiogenic effect of TFRD on Human Umbilical Vein Endothelial Cells (HUVECs) was observed. In vivo, a rat model of Achilles tendon–bone injury was established and animals were divided into four groups: SHAM, Model, BMSCs, and BMSCs + TFRD. After an 8-week intervention, the level of functional recovery was evaluated through histological analysis, immunohistochemistry, serum biochemical analysis, and biomechanical testing. (3) Results: A concentration of 5.0 μg/mL TFRD significantly promoted the proliferation, migration, and differentiation of BMSCs and enhanced the tube formation capacity of HUVECs. In the BMSCs + TFRD group, histological analysis revealed well-organized collagen fibers, increased cartilage deposition, and an optimized tendon–bone interface (TBI) structure. Immunohistochemistry showed upregulated expression of COL I, COL II, and SOX-9, alongside downregulated VEGFA. Furthermore, serum IL-6 levels were decreased, while IL-10 and TGF-β levels were elevated. The biomechanical properties were also significantly improved in this group. (4) Conclusions: TFRD promotes tendon–bone healing and functional recovery by enhancing BMSC functions, promoting angiogenesis, and improving the local microenvironment.

## Simple Summary

Tendon–bone injuries are common in sports and often lead to poor recovery due to insufficient healing at the tendon–bone interface. Current surgical treatments can restore structure but rarely achieve full functional recovery. In this study, we explored how total flavonoids of *Rhizoma drynariae* (TFRD), a natural extract widely used in traditional Chinese medicine, can enhance the regenerative capacity of bone marrow mesenchymal stem cells (BMSCs) for tendon–bone repair. Through both cell experiments and a rat Achilles tendon injury model, we found that TFRD significantly promoted BMSC proliferation, migration, and differentiation, while also stimulating blood vessel formation and reducing local inflammation. When combined with BMSCs, TFRD improved collagen alignment, cartilage formation, and mechanical strength at the healing site. These findings provide experimental evidence that combining TFRD with BMSCs may represent an effective and safe therapeutic strategy for promoting tendon–bone interface regeneration and accelerating functional recovery after sports injuries.

## 1. Introduction

Tendon–bone injuries represent both a major challenge and a prominent focus in the field of sports-related trauma. Globally, over 30 million cases of tendon–bone injuries are reported annually, predominantly involving rotator cuff tears, anterior cruciate ligament (ACL) injuries, and Achilles tendon ruptures [1]. Clinically, such injuries often manifest as pain, swelling, and restricted joint mobility, significantly compromising patients’ quality of life and occupational performance and, in some cases, leading to psychological complications such as anxiety and depression. If left untreated, these injuries may progress to chronic pain, recurrent injuries, and long-term functional impairments [2].

Conventional therapeutic approaches for tendon–bone injuries are limited in efficacy. Currently, surgical reconstruction using tendon grafts placed into bone tunnels remains the standard of care. However, postoperative healing at the tendon–bone interface (TBI) is typically characterized by fibrous scar tissue formation, which lacks the biomechanical strength of native tendon–bone attachments. This results in suboptimal functional recovery and a high rate of re-tear, reported to range between 26% and 94% [3]. One of the major factors contributing to poor healing outcomes is inadequate vascularization at the TBI site, which delays the healing process [4]. Insufficient angiogenesis restricts oxygen and nutrient supply, reduces the survival of newly formed cells, and ultimately impairs tissue regeneration [5].

To address these limitations, various strategies have been explored to enhance TBI healing, including the use of biomaterials, platelet-rich plasma (PRP), cytokines, stem cells, extracellular vesicles, and physical therapies [6,7,8,9,10,11]. Among these, bone marrow mesenchymal stem cells (BMSCs) have emerged as a promising therapeutic candidate in tendon–bone regeneration [12,13]. BMSCs offer several advantages due to their multipotent differentiation capacity, paracrine activity, and regenerative potential. These cells can differentiate into osteoblasts, chondrocytes, and fibroblasts, contributing to collagen deposition and fibrocartilage formation. In addition, BMSCs secrete pro-angiogenic and anti-inflammatory factors that support the local microenvironment and promote healing [1,14].

Despite their potential, BMSC-based therapies face several challenges. The functional performance of BMSCs can be compromised by the hostile microenvironment and insufficient vascularization at the injury site, leading to reduced cell viability and therapeutic efficacy [15,16]. Furthermore, the differentiation of BMSCs is tightly regulated by local cues, and BMSCs alone may not reliably differentiate into the specific cell types required for optimal TBI regeneration. Therefore, strategies to improve the local microenvironment, enhance cell survival, and direct BMSC differentiation toward osteochondral lineages are critical for the success of BMSC-based therapies in tendon–bone repair.

TFRD is a group of natural flavonoid compounds extracted from the dried rhizome of *Drynaria fortunei* (Polypodiaceae). It represents the major bioactive component responsible for the multiple pharmacological effects of *Rhizoma drynariae* [17]. At present, TFRD has been widely applied in both experimental and clinical studies, and has been successfully developed into traditional Chinese patent medicines such as Qianggu Capsules. In traditional Chinese medicine theory, *Rhizoma drynariae* is renowned for its properties of “tonifying the kidney, strengthening the bone, promoting blood circulation, and relieving pain.” It is commonly prescribed for conditions such as lumbar soreness due to kidney deficiency, osteoporosis, fractures, and traumatic injuries, with these therapeutic effects primarily attributed to its rich total flavonoid content [18]. Modern pharmacological studies have demonstrated that TFRD and its monomeric constituents exert a wide range of biological activities, including neuroprotective, antioxidant, anti-osteoporotic, anti-inflammatory, and bone-repair-promoting effects [19]. In addition, pharmacokinetic investigations have revealed that the major active flavonoid constituents of TFRD, such as naringin, neoeriocitrin, and luteolin, are rapidly absorbed in the small intestine, undergo extensive phase II metabolism (mainly glucuronidation and sulfation), and are distributed predominantly in bone, liver, and kidney tissues. These compounds exhibit relatively low oral bioavailability due to first-pass metabolism but achieve sustained plasma exposure through enterohepatic circulation, which supports their long-term osteogenic and anti-inflammatory effects [20].

TFRD has been demonstrated to significantly promote BMSC proliferation and induce osteogenic and chondrogenic differentiation, providing a cellular foundation for tissue regeneration [21,22]. In addition, TFRD enhances angiogenesis by stimulating the proliferation and migration of human umbilical vein endothelial cells (HUVECs), facilitating neovascularization and improving nutrient and oxygen delivery to the injury site [23]. Moreover, TFRD can attenuate local inflammation by suppressing pro-inflammatory cytokine expression, further optimizing the regenerative microenvironment [24].

Collectively, these properties suggest that TFRD can not only augment the proliferative and differentiation capacity of BMSCs but also modulate the local microenvironment and angiogenic response to support tissue repair. This study, therefore, aims to investigate the synergistic effect of TFRD and BMSCs in tendon–bone healing, addressing the limitations of BMSC monotherapy and providing experimental evidence for a more effective therapeutic strategy. The findings are expected to lay a scientific foundation for future research and potential clinical applications in TBI repair.

## 2. Materials and Methods

### 2.1. Materials and Equipment

All experimental animals used in this study were procured from the Laboratory Animal Center of Southern Medical University (License No. SCXK [Yue] 2021-0041), and all procedures were approved by the Ethics Committee of Guangzhou Sport University (Approval No. 2023LCLL-87). All animal experiments were conducted in strict accordance with institutional and national guidelines for the care and use of laboratory animals.

The key reagents and instruments used in this study included the CCK-8 assay kit (Beijing Dingguosheng Biotechnology Co., Ltd., Beijing, China), osteogenic and chondrogenic induction kits for rat BMSCs, and angiogenesis induction medium for HUVECs (Saiye Biotechnology Co., Ltd., Guangzhou, China), Matrigel matrix gel (Corning Life Sciences, Wujiang, China), CD29-APC and CD90-PE-CY7 antibodies (Elabscience Biotechnology Co., Ltd., Wuhan, China), CD34-PE antibody (Bioss Biotechnology Co., Ltd., Beijing, China), Qianggu capsules (Beijing Qihuang Pharmaceutical Co., Ltd., Beijing, China; Batch/Lot No.: Z20030007), hematoxylin–eosin (H & E) staining solution, Masson’s trichrome staining kit, Safranin O–Fast Green staining kit (BioS Biotech Co., Ltd., Hubei, China), ELISA kits (Kedi Yunxiang Biotech Co., Ltd., Nanjing, China), cell migration analysis plug-in (Shenzhen Kuyuan Biotech Co., Ltd., Shenzhen, China), and a microcomputer-controlled universal testing machine (Bairo Testing Equipment Co., Ltd., Shanghai, China).

### 2.2. Isolation and Identification of BMSCs

**Figure 1:**
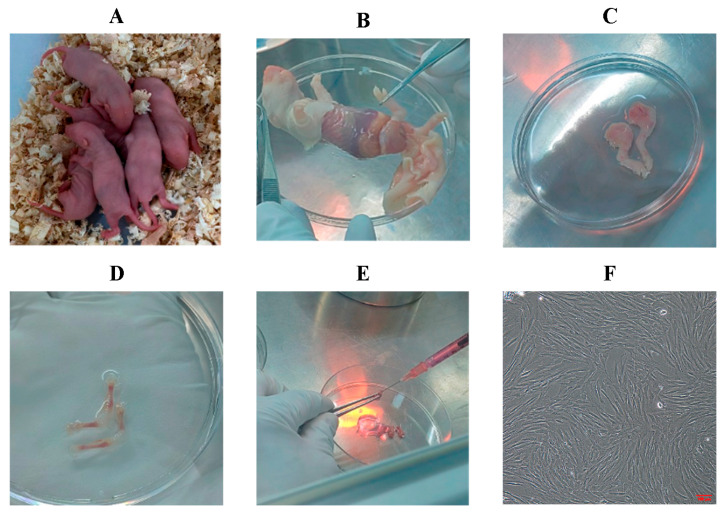
Isolation procedure of primary rat BMSCs. (**A**) 5–7-day-old rats; (**B**) Removal of skin; (**C**) Dissection of hind limbs; (**D**) Isolation of tibia and femur; (**E**) Flushing of bone marrow cavity; (**F**) Purified P3 BMSCs (100×).

Isolation of BMSCs: ① Five- to seven-day-old Sprague-Dawley (SD) rats (Figure 1A) were euthanized by cervical dislocation and immersed in 75% ethanol for 5 min for sterilization. ② The skin was removed (Figure 1B), and both hind limbs were dissected by cutting along the superior border of the hip bone (Figure 1C). ③ Muscle tissue was carefully removed, leaving only the tibia and femur (Figure 1D), which were then transferred to a Petri dish containing PBS and rinsed two to three times. ④ A surgical blade was used to cut open both ends of the femur and tibia to expose the bone marrow cavity. ⑤ The bone marrow cavity was flushed with 1 mL of rat Bone Marrow Mesenchymal Stem Cell-Complete Medium (BMMSC-CM) until the bone appeared white (Figure 1E). ⑥ The flushed bone marrow suspension was collected into a T25 culture flask and incubated at 37 °C in a 5% CO_2_ humidified incubator. ⑦ The medium was changed for the first time 1–2 days after primary cell culture, and subsequently every 2–3 days. Cells were passaged when they reached 70–80% confluence (Figure 1F, 10×).

BMSC Surface Marker Analysis: ① Passage 3 (P3) rat BMSCs were detached using 0.25% trypsin-EDTA solution. The reaction was stopped with BMMSC-CM, and the cell suspension was collected into a centrifuge tube and counted. ② Cells were centrifuged at 250× *g* for 4 min, and the supernatant was discarded. ③ A single-cell suspension (75 µL, approximately 1 × 10^6^ cells) was added to each of two flow cytometry tubes. One tube served as a negative control, and the other as the experimental tube. To the experimental tube, 5 µL of CD29 APC, 5 µL of CD90 PE-Cy7, and 1 µL of CD34 PE antibodies were added. ④ The tubes were incubated at 4 °C in the dark for 1 h. ⑤ Two mL of PBS was added to each tube, followed by centrifugation at 250× *g* for 5 min. The supernatant was removed, and the cells were resuspended in 200 µL of PBS for flow cytometry analysis.

### 2.3. Effect of TFRD on Cell Proliferation

Two cell types were used in this assay: BMSCs and HUVECs. Cells were divided into four groups: Control group (complete medium without TFRD), low-dose TFRD group (1.0 mg/L), medium-dose TFRD group (5.0 mg/L), and high-dose TFRD group (10.0 mg/L), with TFRD supplemented into the respective complete media. Each group was set up with three replicate wells.

At 12, 24, and 48 h after TFRD intervention, cell viability was assessed using the CCK-8 assay. Wells containing only medium and CCK-8 solution were used as the blank control. Absorbance at 450 nm was measured using a microplate reader. Cell viability (%) was calculated using the following formula: Cell viability (%) = [(OD_t_ − OD_blank)/(OD_control − OD_blank)] × 100.

### 2.4. Effect of TFRD on Cell Migration

This experiment involved two cell types: BMSCs and HUVECs. The cells were divided into two groups: a Control group, cultured in medium containing 2% fetal bovine serum (FBS) but without TFRD, and a TFRD group, cultured in the same medium supplemented with 5.0 µg/mL TFRD. This concentration was selected based on its optimal proliferative effect on both BMSCs and HUVECs, as determined in the experiments described in Section 3.1.2 and Section 3.2.1. Each group was prepared in triplicate.

For the scratch assay, 70 μL of P3 BMSCs at a concentration of 5 × 10^5^/mL were seeded into wells equipped with a cell scratch insert. When cells reached 90% confluence, the insert was carefully removed to create a standardized cell-free gap (scratch). Cells were then cultured in the respective media. Migration was monitored at 0 h, 12 h, and 24 h post-intervention under an inverted microscope. The migration rate was calculated as: Migration rate (%) = (Initial scratch area − Scratch area at time t)/Initial scratch area × 100%.

### 2.5. Effect of TFRD on Osteogenic Differentiation of BMSCs

The experimental groups included: a Control group, cultured in complete osteogenic induction medium without TFRD, and a TFRD group, cultured in the same medium supplemented with 5.0 µg/mL TFRD. This concentration was previously identified as optimal for BMSC proliferation in experiments described in Section 3.1.2. Each group was prepared in triplicate wells.

Cells were seeded in gelatin-coated six-well plates at a density of 2 × 10^4^ cells/cm^2^. Once they reached approximately 70% confluence, the respective osteogenic media were added according to group allocation. The medium was refreshed every 3 days. The differentiation process was observed daily under a microscope, and Alizarin Red staining was performed when calcium nodules became visibly prominent.

### 2.6. Effect of TFRD on Chondrogenic Differentiation of BMSCs

The experiment was divided into two groups: a Control group, cultured in complete chondrogenic induction medium without TFRD, and a TFRD experimental group, cultured in the same induction medium supplemented with 5.0 µg/mL TFRD. This concentration was selected based on its optimal proliferative effect on BMSCs, as determined by the results in Section 3.1.2. Each group was established with three replicates. A total of 4 × 10^5^ cells were transferred into 15 mL conical tubes and pelleted according to the manufacturer’s instructions provided with the chondrogenic differentiation kit. After cell aggregation, the chondrogenic pellets were gently dislodged from the tube bottom to float freely in the medium. The culture medium was replaced every 2–3 days. When the cartilage spheroids reached a diameter of approximately 1.5–2 mm, Alcian Blue staining was performed to assess matrix production.

### 2.7. Effect of TFRD on Angiogenesis in HUVECs

The experimental groups included: a Control group, cultured in angiogenic induction medium without TFRD, and a TFRD group, cultured in the same medium supplemented with 5.0 µg/mL TFRD. This concentration was previously identified as optimal for HUVEC proliferation in experiments described in Section 3.2.1. Each group was prepared in triplicate wells. HUVECs were seeded in culture flasks and allowed to grow until 60–70% confluence. The medium was then replaced with angiogenesis induction medium and incubated overnight. Matrigel (pre-cooled on ice) was added to each well of a 12-well plate and allowed to solidify at 37 °C. Subsequently, 1 × 10^6^ HUVECs were seeded per well onto the Matrigel, with TFRD added to the experimental group. After 5 h of incubation, tube formation was assessed and photographed under a microscope.

### 2.8. Animal Grouping and Interventions

A total of sixty 8-week-old male Sprague-Dawley (SD) rats, weighing 220 ± 20 g, were randomly allocated into four groups (n = 15 per group) using a computer-generated random number table: the Sham group (Sham), the Model group, the BMSC treatment group (BMSCs), and the TFRD + BMSC treatment group (TFRD + BMSCs). A rat Achilles tendon–calcaneus insertion injury model was established based on previously described protocols [25]. SHAM rats underwent only a longitudinal skin incision without tendon or bone manipulation to control for surgical stress. In the other three groups, unilateral injury to the left Achilles tendon–calcaneus junction was induced.

**Figure 2:**
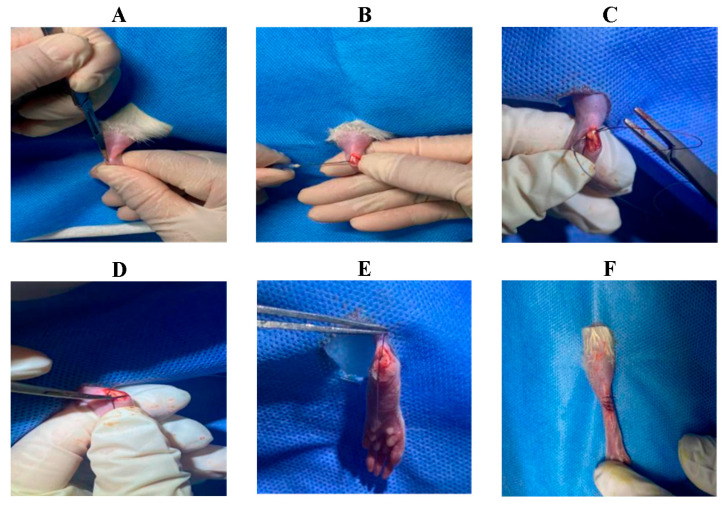
Surgical procedure for creating the rat Achilles tendon injury model. (**A**) Longitudinal incision; (**B**) Creation of bone tunnel; (**C**) Circumferential suture placement around the Achilles tendon; (**D**) Transection of the Achilles tendon at its insertion (TBI); (**E**) Reattachment of the Achilles tendon to the calcaneus; (**F**) Skin closure.

Achilles Tendon-Calcaneus Injury Model Creation: ① The surgical site (left hind limb) was shaved and sterilized (Supplementary Figures S1–S3). ② A longitudinal incision approximately 4 mm in length was made approximately 2 mm proximal to the calcaneus using a scalpel to expose the calcaneus and Achilles tendon (Figure 2A). ③ A transverse bone tunnel was created in the calcaneus using a 21-gauge needle (Figure 2B). ④ A 3-0 absorbable suture was passed through the calcaneal bone tunnel. The needle was then passed obliquely through the Achilles tendon from plantar to dorsal, approximately 2 mm proximal to the insertion point, and exited through the dorsal tendon surface. Next, the needle was passed back through the tendon from the opposite side in a plantar-to-dorsal direction, creating a circumferential loop around the tendon. Finally, the needle was passed through the tendon from plantar to dorsal, just distal to the initial entry point, completing the tendon fixation (Figure 2C). ⑤ The Achilles tendon was transected at its insertion point using surgical scissors, and any remaining soft tissue at the tendon–bone interface was carefully removed using a scalpel (Figure 2D). ⑥ Finally, the suture ends from the tendon fixation were tied to the suture exiting the bone tunnel, reattaching the Achilles tendon to the calcaneus (Figure 2E). The skin was closed in layers using a new suture (Figure 2F).

At 24 h post-operation, both the BMSCs group and the TFRD + BMSCs group received a local injection of 0.2 mL BMSCs suspension (5 × 10^6^ cells/mL) at the injury site [14]. All groups received daily oral gavage to ensure consistent administration routes. Specifically, the BMSCs group and the Model group were administered 0.9% sterile normal saline (3 mL/kg/day), while the TFRD + BMSCs group received TFRD dissolved in the same 0.9% sterile saline at a dose of 80 mg/kg/day [26].

### 2.9. Histological Staining

After 8 weeks of intervention, six animals were randomly selected from each group and euthanized. At the end of the 8-week intervention, rats were euthanized humanely. The left hind limbs were dissected, and muscle tissue was carefully removed to preserve tissue samples from the mid-calf to the midfoot region. Samples were fixed in 10% neutral-buffered formalin for at least 24 h. After fixation, tissues were embedded in paraffin and longitudinally sectioned along the Achilles tendon axis at a thickness of 10 µm. The sections were then dewaxed, rehydrated, and subjected to standard histological protocols, including hematoxylin and eosin (H & E) staining, Masson’s trichrome staining, Safranin O–Fast Green (SO/FG) staining, and immunohistochemical (IHC) staining.

### 2.10. Assessment of Tendon Maturation

Tendon maturation was evaluated using a previously established scoring system for histological assessment of tendon development [27], based on the stained sections described above.

### 2.11. Serum Biochemical Analysis

Eight weeks post-intervention, six rats were randomly selected from each group. At week 8, rats were lightly anesthetized and fixed in a supine position. The fur over the clavicular region was shaved to expose the jugular vein. Approximately 1 mL of venous blood was collected using a syringe and transferred into lithium heparin anticoagulant tubes. After resting at room temperature for 2 h, the blood samples were centrifuged at 3000 rpm for 10 min to obtain serum. Serum levels of IL-6, IL-10, and TGF-β were quantified using enzyme-linked immunosorbent assay (ELISA) kits.

### 2.12. Biomechanical Testing of the Achilles Tendon–Calcaneus Interface

At 8 weeks post-intervention, six rats from each group were randomly selected for biomechanical testing. The animals were euthanized, and the left hind limb was harvested. Muscle tissue was carefully removed, leaving the Achilles tendon–calcaneus complex intact for testing.

**Figure 3:**
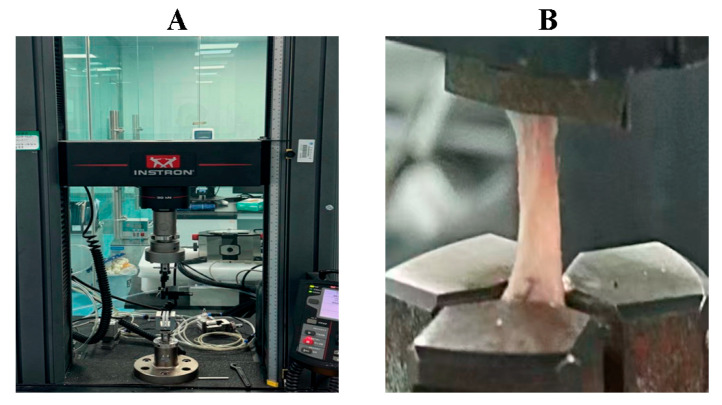
Biomechanical testing of the rat Achilles tendon–calcaneus interface using a microcomputer-controlled universal testing machine. (**A**) Microcomputer-controlled universal testing machine; (**B**) Tensile testing procedure of the Achilles tendon–calcaneus junction.

Ultimate tensile load was measured using a microcomputer-controlled universal testing machine (Figure 3). The Achilles tendon–calcaneus specimens were mounted between the upper and lower clamps of the biomechanical device. The samples were subjected to axial tension along the long axis of the tendon–bone interface at a constant speed of 0.1 mm/s. The maximum force at the point of failure was recorded as the ultimate tensile load of the interface.

### 2.13. Statistical Analysis

All statistical analyses were performed using SPSS software (version 26.0). Normality and homogeneity of variances were tested using the Shapiro–Wilk test and Levene’s test, respectively. Normally distributed data were analyzed by one-way ANOVA, whereas non-normally distributed data were analyzed using the Kruskal–Wallis test. Differences were considered statistically significant at *p* < 0.05. Image analysis was carried out with Fiji-ImageJ 1.54, and figures were plotted using GraphPad Prism 10.0.

## 3. Results

### 3.1. TFRD Enhances the Osteogenic and Chondrogenic Differentiation Potential of BMSCs In Vitro

#### 3.1.1. Isolation and Characterization of Rat BMSCs

**Figure 4:**
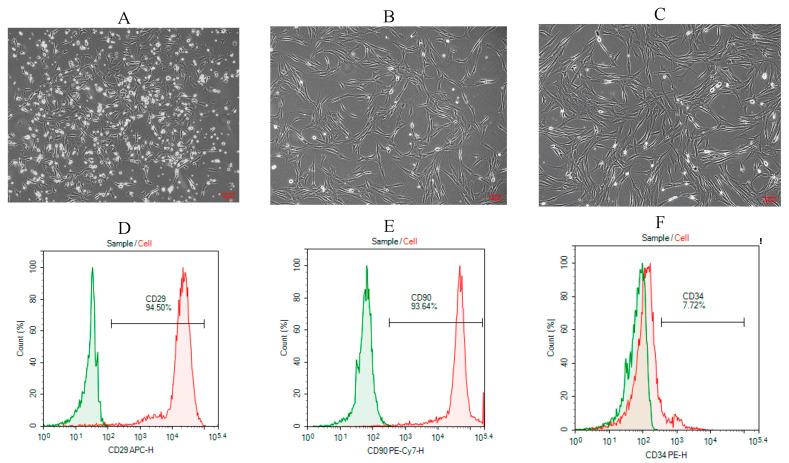
Morphological observation and flow cytometric characterization of rat BMSCs. (**A**) Primary (P0) BMSCs showed granular colony-like clusters (100×); (**B**) Passage 1 (P1) BMSCs exhibited short spindle-shaped adherent growth (100×); (**C**) Passage 3 (P3) BMSCs displayed elongated spindle-shaped morphology with adherent growth (100×); (**D**) Flow cytometric analysis of CD29 expression; (**E**) CD90 expression; (**F**) CD34 expression.

Rat BMSCs were isolated and purified using the whole bone marrow adherence method. In primary culture, BMSCs appeared as oval or spherical colonies adherent to the culture surface (Figure 4A). At passage 1, cells gradually became more uniformly distributed, exhibiting a vortex-like growth pattern, with some displaying spindle-shaped morphology (Figure 4B). By passage 3, the cells showed a regular, uniform distribution and adhered in a long spindle-like shape (Figure 4C). Flow cytometric analysis demonstrated positive expression of CD29 and CD90 at rates of 94.5% and 93.64%, respectively (Figure 4D,E), while CD34 expression was only 7.72% (Figure 4F). These results confirm that the isolated cells possessed characteristic surface markers of mesenchymal stem cells. According to the minimal criteria proposed by the International Society for Cellular Therapy (ISCT), mesenchymal stem cells are defined as plastic-adherent cells with trilineage differentiation potential that express CD29 and CD90 but lack hematopoietic markers such as CD34 [28]. The expression profile obtained in this study is therefore consistent with the ISCT standard for mesenchymal stem cell identification.

#### 3.1.2. Optimal Concentration Analysis of TFRD for Promoting BMSC Proliferation

**Figure 5:**
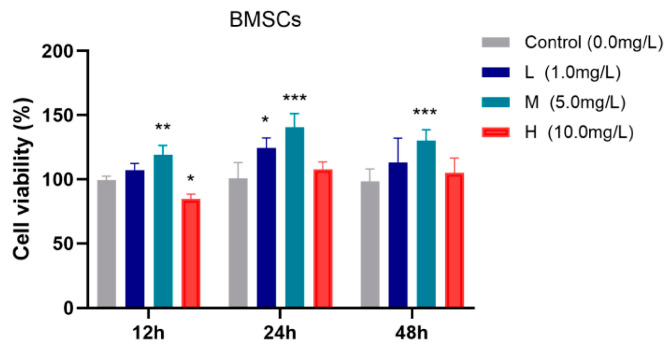
Effects of different concentrations of TFRD on the proliferative activity of BMSCs. L: low-dose group; M: medium-dose group; H: high-dose group. * *p* < 0.05, ** *p* < 0.01, *** *p* < 0.001 vs. Control group.

The effects of different concentrations of TFRD on BMSC viability were evaluated using the CCK-8 assay, and the results are presented in Figure 5.

After 12 h of incubation, the medium-dose TFRD group exhibited significantly higher cell viability compared to the Control group (*p* < 0.01), whereas the high-dose group showed a slight reduction in viability (*p* < 0.05). At 24 h, both the low-dose (*p* < 0.05) and medium-dose (*p* < 0.001) groups demonstrated significantly increased cell viability relative to the Control group. After 48 h, the medium-dose group continued to show a significant enhancement in cell viability (*p* < 0.001).

In summary, the medium-dose TFRD group consistently promoted BMSC viability at all observed time points (12 h, 24 h, and 48 h), indicating an optimal proliferative effect. Therefore, the medium concentration of TFRD was selected for use in all subsequent experiments.

#### 3.1.3. TFRD Promotes the Migration of BMSCs

**Figure 6:**
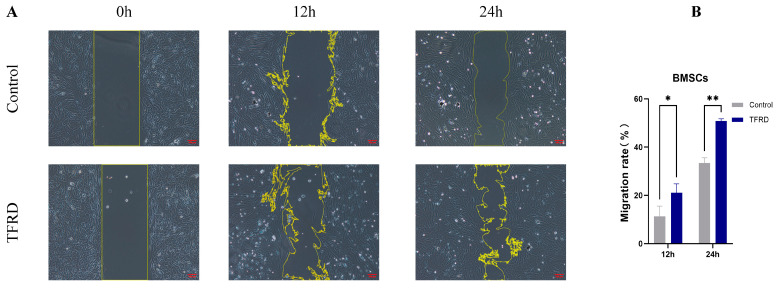
Effect of TFRD on the migratory capacity of BMSCs. (**A**) Representative images showing cell migration in the Control and TFRD groups at 0 h, 12 h, and 24 h (100× magnification); (**B**) Quantitative comparison of migration rates between the Control and TFRD groups. * *p* < 0.05; ** *p* < 0.01.

The effect of TFRD on the migratory capacity of BMSCs was assessed using a scratch wound assay. As shown in Figure 6A, there was no significant difference in scratch width between the TFRD and Control groups at 0 h. However, at 12 h and 24 h post-treatment, BMSCs in the TFRD group exhibited noticeably faster wound closure compared to the Control group. Cells at the scratch edge in the TFRD group displayed more elongated and polarized morphologies, with prominent lamellipodia formation, indicating enhanced migratory activity. Semi-quantitative analysis of the migration rate (Figure 6B) revealed that at 12 h, the TFRD-treated group showed a significantly higher migration rate compared to the Control group (*p* < 0.05), and this difference became more pronounced at 24 h (*p* < 0.01). These findings demonstrate that TFRD significantly enhances the migration ability of BMSCs in vitro.

#### 3.1.4. TFRD Promotes Osteogenic and Chondrogenic Differentiation of BMSCs In Vitro

**Figure 7:**
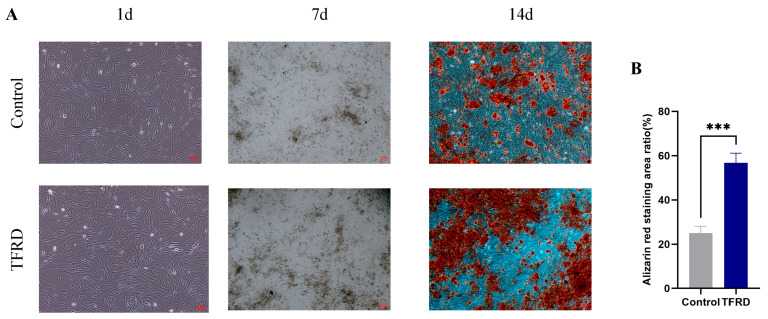
Effect of TFRD on the osteogenic differentiation of BMSCs. (**A**) Representative images of osteogenic differentiation in the Control and TFRD groups on day 1 (100×), day 7 (40×), and day 14 (100×) of induction; (**B**) Comparison of calcium nodule formation between the Control and TFRD groups on day 14. *** *p* < 0.001.

The effect of TFRD on osteogenic differentiation of BMSCs was investigated using an osteogenic induction assay. As shown in Figure 7A, at day 1 of osteogenic induction, both groups exhibited typical mesenchymal stem cell morphology with no apparent signs of osteogenic differentiation. By day 7, mineralized nodules were observed in both groups, with a greater number of nodules present in the TFRD group compared to the control group, suggesting that TFRD treatment accelerated the osteogenic differentiation process. At day 14, prominent mineralized nodules were evident in both groups.

Semi-quantitative analysis of the mineralized nodules at day 14 (Figure 7B) revealed a significantly larger area of calcium nodule staining in the TFRD group compared to the control group (*p* < 0.001). These results demonstrate that TFRD treatment significantly promotes osteogenic differentiation of BMSCs.

**Figure 8:**
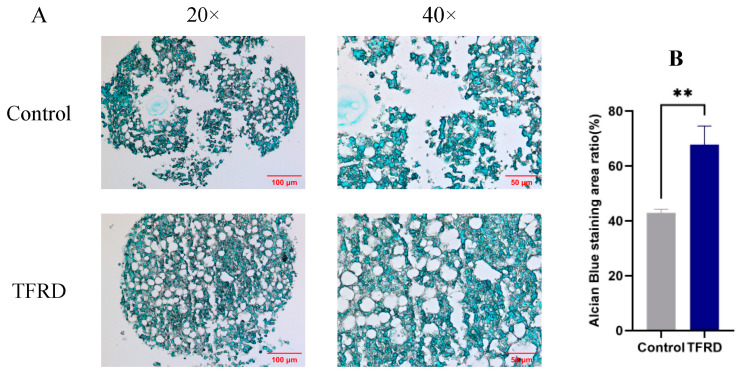
Effect of TFRD on chondrogenic differentiation of BMSCs. (**A**) Representative images of chondrogenic pellets from the control and TFRD groups at day 21 of chondrogenic induction (20×, 40×). (**B**) Comparison of cartilage matrix in the control and TFRD groups at day 21. ** *p* < 0.01.

As shown in Figure 8A, after 21 days of chondrogenic induction, both groups successfully formed cartilage pellets. Under 20× magnification, the control group pellets exhibited a loose structure with pale and uneven staining, indicating a lower content or uneven distribution of sulfated glycosaminoglycans (GAGs). This resulted in a less compact structure with apparent disruptions. In contrast, the TFRD group displayed compact pellets with significantly larger areas of more intense and uniform staining, suggesting enhanced cartilage matrix deposition. At 40× magnification, the TFRD group exhibited a more continuous cartilage matrix with fewer gaps, in stark contrast to the control group. This suggests that TFRD treatment optimizes the cartilage matrix architecture, promoting cartilage tissue formation.

Semi-quantitative analysis of the cartilage matrix (Figure 8B) revealed a significantly higher Alcian blue staining area (representing cartilage matrix quantity) in the TFRD group compared to the control group (*p* < 0.01). These results demonstrate that TFRD significantly promotes chondrogenic differentiation of BMSCs.

These in vitro results demonstrate that TFRD significantly enhances the osteogenic and chondrogenic differentiation potential of BMSCs, providing a cellular and functional foundation for the tissue-level regeneration observed in the subsequent in vivo experiments (Section 3.3).

### 3.2. TFRD Promotes Angiogenesis to Enhance Tissue Regeneration

#### 3.2.1. Determination of the Optimal Concentration of TFRD for Promoting HUVEC Proliferation

**Figure 9:**
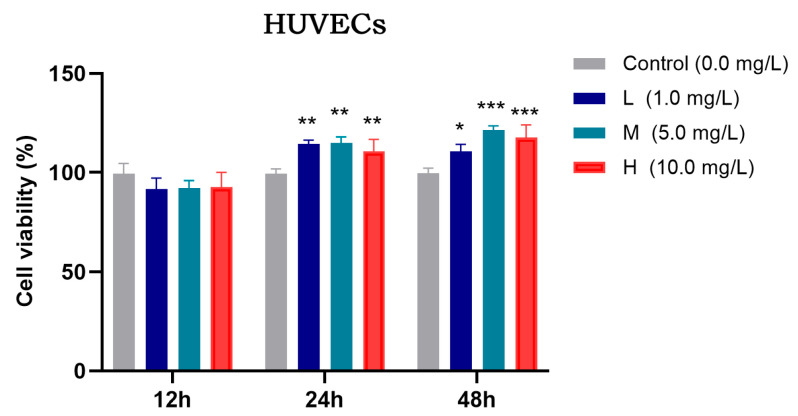
Effects of different concentrations of TFRD on the proliferative activity of HUVECs. L: low concentration; M: medium concentration; H: high concentration. * *p* < 0.05; ** *p* < 0.01; *** *p* < 0.001 vs. Control group.

The proliferative activity of HUVECs under different concentrations of TFRD was assessed using the CCK-8 assay, and the results are shown in Figure 9. After 12 h of incubation, there was no significant difference in cell viability among the experimental groups compared to the Control group (*p* > 0.05). At 24 h, however, all experimental groups (low-, medium-, and high-dose) exhibited significantly increased proliferation compared to the Control group (*p* < 0.01). By 48 h, cell viability in the low-dose group was significantly higher than the Control group (*p* < 0.05), while both the medium- and high-dose groups showed even more pronounced increases (*p* < 0.001).

In summary, both medium and high concentrations of TFRD effectively promoted HUVEC proliferation. However, considering pharmacological appropriateness, the previously established optimal concentration for BMSC modulation, and the need to avoid potential adverse effects associated with higher doses, the medium concentration of TFRD was selected for subsequent experiments. This concentration not only effectively enhances HUVEC proliferation but also aligns with the optimal dosage for BMSCs, ensuring consistency and synergy in subsequent co-culture and mechanistic studies.

#### 3.2.2. TFRD Enhances the Migration of Endothelial Cells

**Figure 10:**
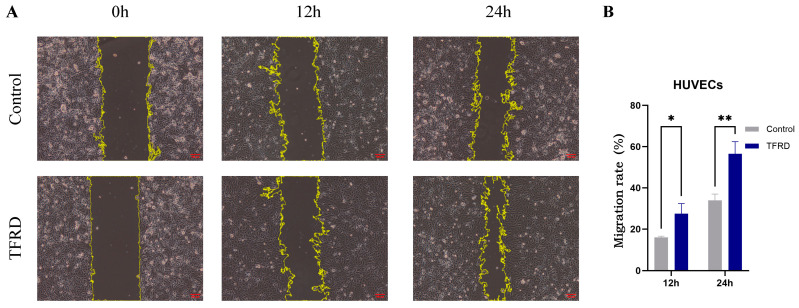
Effect of TFRD on the migratory capacity of HUVECs. (**A**) Representative images showing cell migration in the Control and TFRD groups at 0 h, 12 h, and 24 h (100× magnification); (**B**) Comparison of cell migration rates between the Control and TFRD groups. * *p* < 0.05; ** *p* < 0.01.

The effect of TFRD on the migratory capacity of HUVECs was evaluated using a scratch wound assay. As shown in Figure 10A, at 0 h, there was no difference in scratch width between the Control and TFRD groups, indicating a consistent starting point. By 12 h, the TFRD group exhibited noticeably faster migration, with a marked reduction in scratch width compared to the Control group, suggesting that TFRD promoted HUVEC migration. At 24 h, the scratch area in the TFRD group had nearly closed, whereas the Control group still exhibited a considerable gap, further demonstrating the enhanced migratory ability induced by TFRD.

Semi-quantitative analysis of the migration rate (Figure 10B) showed that at 12 h, the TFRD group had a significantly higher migration rate than the Control group (*p* < 0.05), and this difference became highly significant at 24 h (*p* < 0.01). These findings indicate that TFRD exerts a significant pro-migratory effect on HUVECs.

#### 3.2.3. TFRD Enhances Tissue Regeneration by Promoting Angiogenesis

**Figure 11:**
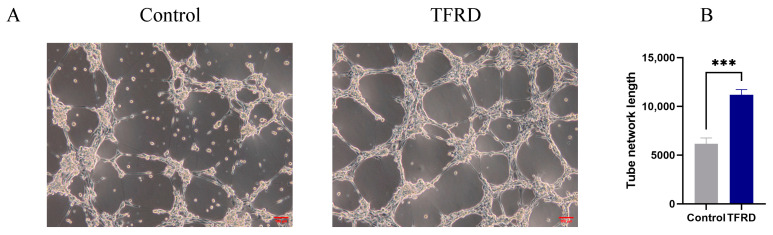
Effect of TFRD on the Vasculogenic Differentiation of HUVECs. (**A**) Representative images of tube formation in the Control and TFRD groups; (**B**) Quantitative comparison of total tube length between the Control and TFRD groups. *** *p* < 0.001.

The pro-angiogenic effect of TFRD on HUVECs was evaluated using an endothelial tube formation assay. As shown in Figure 11A, the vascular-like structures formed by cells in the Control group were sparse, with an incomplete tubular network and fewer branching points and tubular structures. In contrast, cells in the TFRD-treated group exhibited a markedly denser and more continuous tubular network, with a significant increase in both the number of branching points and the integrity of the tubular connections.

Further semi-quantitative analysis of total tube length demonstrated that the vascular length in the TFRD group was significantly greater than that in the Control group, with highly significant statistical differences (*p* < 0.01) (Figure 11B). These findings indicate that TFRD exerts a pronounced promotive effect on angiogenesis in HUVECs.

### 3.3. TFRD Enhances BMSC-Mediated Tendon–Bone Healing in Rats

#### 3.3.1. TFRD Enhances BMSC-Mediated Tendon–Bone Tissue Regeneration

**Figure 12:**
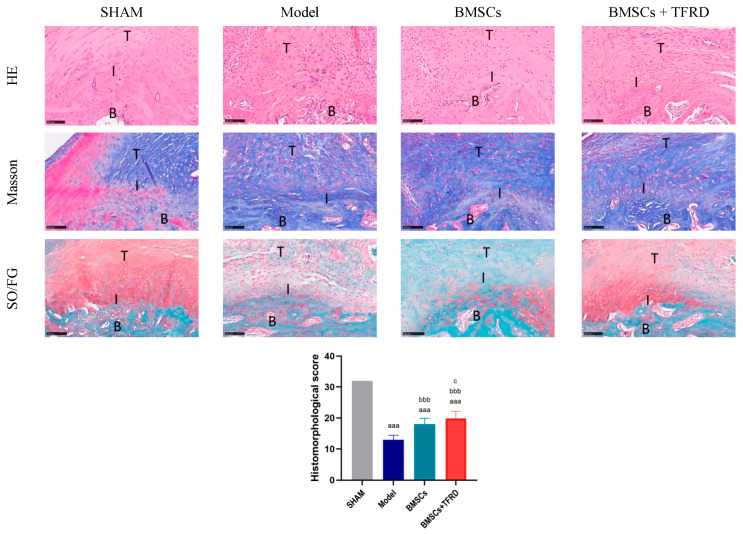
Histological evaluation of the Achilles tendon–calcaneus interface in different experimental groups at 8 weeks post-operation using H & E, Masson’s trichrome, and SO/FG staining, along with histomorphological scoring. T: tendon; I: tendon–bone interface; B: bone. Scale bar = 100 μm. ^aaa^ *p* < 0.001 vs. SHAM group; ^bbb^ *p* < 0.001 vs. Model group; ^c^ *p* < 0.05 vs. BMSCs group.

At 8 weeks post-intervention, tissue regeneration at the TBI was evaluated using H & E, Masson’s trichrome, and SO/FG staining, along with histomorphological scoring (Figure 12).

H & E staining showed that the SHAM group presented a well-organized TBI structure, with densely packed and aligned collagen fibers and a distinct fibrocartilaginous transition zone. In contrast, the Model group exhibited disorganized structure, loss of the transition zone, and irregular collagen fiber orientation. In the BMSCs group, partial restoration of the transitional structure and improved—but still discontinuous—collagen alignment were observed. The BMSCs + TFRD group demonstrated the most complete structural restoration, with well-organized collagen fibers and a clearly defined transition zone, indicating the best repair outcome.

Masson’s staining revealed dense and regularly aligned collagen in the SHAM group, whereas the Model group showed disorganized collagen overgrowth and randomly distributed fibroblasts. The BMSCs group exhibited partial improvement in collagen remodeling, with more orderly fiber arrangement. In the BMSCs + TFRD group, collagen fibers were more uniformly distributed, well-aligned, and fibroblasts were arranged along the fiber bundles, indicating superior tissue organization.

SO/FG staining results showed that the cartilage layer in the SHAM group was structurally intact with abundant matrix. The Model group exhibited a loss of cartilage matrix and sparsely distributed chondrocytes. The BMSCs group showed increased chondrocyte numbers and enhanced matrix deposition. Remarkably, the BMSCs + TFRD group displayed the most pronounced cartilage regeneration, characterized by dense, orderly chondrocyte arrangement, rich matrix, and well-defined structural boundaries, suggesting that the combined treatment significantly promoted cartilage formation and remodeling.

Histological scoring demonstrated that all experimental groups (Model, BMSCs, BMSCs + TFRD) had significantly lower scores than the SHAM group (*p* < 0.001). Compared to the Model group, both the BMSCs and BMSCs + TFRD groups showed significant improvement in histological scores (*p* < 0.001). Notably, the BMSCs + TFRD group achieved significantly higher scores than the BMSCs group alone (*p* < 0.05).

Collectively, these findings indicate that the combined intervention with BMSCs and TFRD markedly improves TBI architecture by promoting collagen fiber alignment, enhancing cartilage matrix deposition, and facilitating overall interface tissue remodeling.

#### 3.3.2. TFRD Enhances the Extracellular Matrix Microenvironment During BMSC-Mediated Tendon–Bone Repair

**Figure 13:**
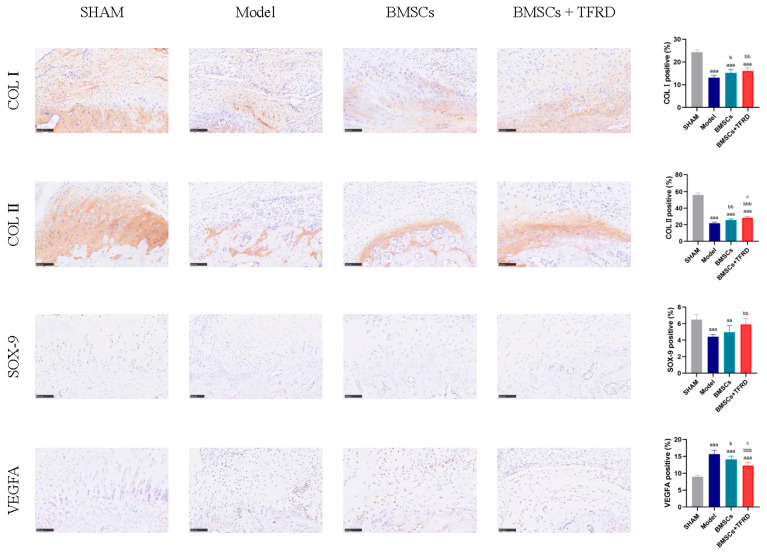
Immunohistochemical staining and semi-quantitative analysis of the Achilles tendon–calcaneus interface in different experimental groups at 8 weeks post-operation. Scale bar = 100 μm. ^aa^ *p* < 0.01, ^aaa^ *p* < 0.001 vs. SHAM group; ^b^ *p* < 0.05, ^bb^ *p* < 0.01, ^bbb^ *p* < 0.001 vs. Model group; ^c^ *p* < 0.05 vs. BMSCs group.

IHC staining of the Achilles tendon–calcaneus interface in different experimental groups is shown in Figure 13.

COL I expression: Compared to the SHAM group, the percentage of COL I-positive staining was significantly reduced in the Model, BMSCs, and BMSCs + TFRD groups (*p* < 0.001). However, relative to the Model group, COL I expression was significantly higher in the BMSCs group (*p* < 0.05) and further elevated in the BMSCs + TFRD group (*p* < 0.01). These results suggest that both BMSCs alone and in combination with TFRD significantly promote COL I synthesis, contributing to tendon matrix reconstruction.

COL II expression: All experimental groups exhibited lower COL II expression compared to the SHAM group (*p* < 0.001). However, COL II positivity in the BMSCs group was significantly higher than that in the Model group (*p* < 0.01), and further increased in the BMSCs + TFRD group (*p* < 0.001). Moreover, the BMSCs + TFRD group showed significantly higher COL II expression than the BMSCs group (*p* < 0.05), indicating a synergistic effect of TFRD on chondrogenic matrix formation.

SOX-9 expression: SOX-9 positivity was significantly reduced in the Model group compared to the SHAM group (*p* < 0.001), and also lower in the BMSCs group (*p* < 0.01). However, the BMSCs + TFRD group exhibited a significant increase in SOX-9 expression compared to the Model group (*p* < 0.01), indicating enhanced chondrogenic transcriptional activation under combined treatment.

VEGFA expression: VEGFA expression was significantly elevated in the Model, BMSCs, and BMSCs + TFRD groups relative to the SHAM group (*p* < 0.001), suggesting a reactive angiogenic response following injury. Compared to the Model group, VEGFA levels were lower in the BMSCs group (*p* < 0.05) and even more reduced in the BMSCs + TFRD group (*p* < 0.01). Additionally, VEGFA expression in the BMSCs + TFRD group was significantly lower than in the BMSCs group (*p* < 0.05), indicating better regulation of neovascularization.

In summary, combined treatment with BMSCs and TFRD more effectively upregulated COL II and SOX-9 expression while mitigating excessive VEGFA elevation compared to BMSCs alone. These results suggest that the BMSCs + TFRD strategy more efficiently optimizes the extracellular matrix microenvironment at the tendon–bone interface, enhances fibrocartilage formation, and fine-tunes vascular regulation, thus exhibiting superior regenerative potential.

#### 3.3.3. TFRD Modulates Cytokine Profiles During BMSC-Mediated Tendon–Bone Healing

**Figure 14:**
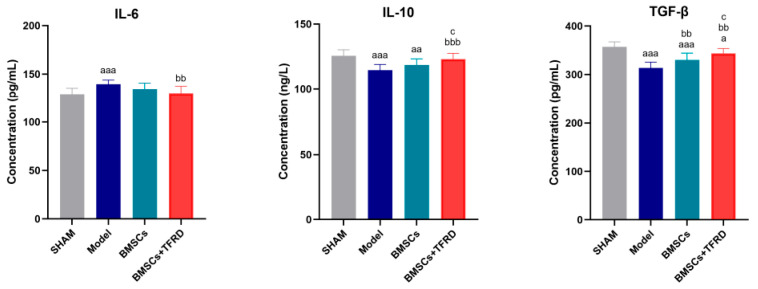
Comparison of serum IL-6, IL-10, and TGF-β levels among different experimental groups at 8 weeks post-operation. ^a^ *p* < 0.05, ^aa^ *p* < 0.01, ^aaa^ *p* < 0.001 vs. SHAM group; ^bb^ *p* < 0.01, ^bbb^ *p* < 0.001 vs. Model group; ^c^ *p* < 0.05 vs. BMSCs group.

At 8 weeks post-intervention, serum levels of IL-6, IL-10, and TGF-β were measured in each experimental group, as shown in Figure 14.

IL-6: The Model group exhibited significantly elevated IL-6 levels compared to the SHAM group (*p* < 0.001), indicating an inflammatory response following injury. In contrast, IL-6 levels in the BMSCs + TFRD group were significantly lower than those in the Model group (*p* < 0.01), suggesting that the combined intervention effectively suppressed inflammation.

IL-10: IL-10 levels were significantly reduced in both the Model (*p* < 0.001) and BMSCs groups (*p* < 0.01) compared to the SHAM group. However, the BMSCs + TFRD group showed a significant increase in IL-10 levels compared to the Model group (*p* < 0.001) and the BMSCs group (*p* < 0.05), indicating an enhanced anti-inflammatory response with TFRD co-administration.

TGF-β: Serum TGF-β levels in the Model and BMSCs groups were markedly lower than in the SHAM group (*p* < 0.001), while the BMSCs + TFRD group also exhibited a reduction, though less pronounced (*p* < 0.05). Compared with the Model group, both BMSCs and BMSCs + TFRD groups had significantly increased TGF-β levels (*p* < 0.01). Furthermore, TGF-β expression was higher in the BMSCs + TFRD group than in the BMSCs group alone (*p* < 0.05).

In summary, both BMSCs and BMSCs + TFRD treatments partially restored the balance of key cytokines involved in inflammation and regeneration. Notably, the BMSCs + TFRD combination more effectively enhanced anti-inflammatory (IL-10, TGF-β) and reduced pro-inflammatory (IL-6) cytokine expression, suggesting superior immunomodulatory capacity and a more favorable microenvironment for tendon–bone interface healing.

#### 3.3.4. TFRD Improves the Biomechanical Properties of BMSC-Mediated Tendon–Bone Repair

**Figure 15:**
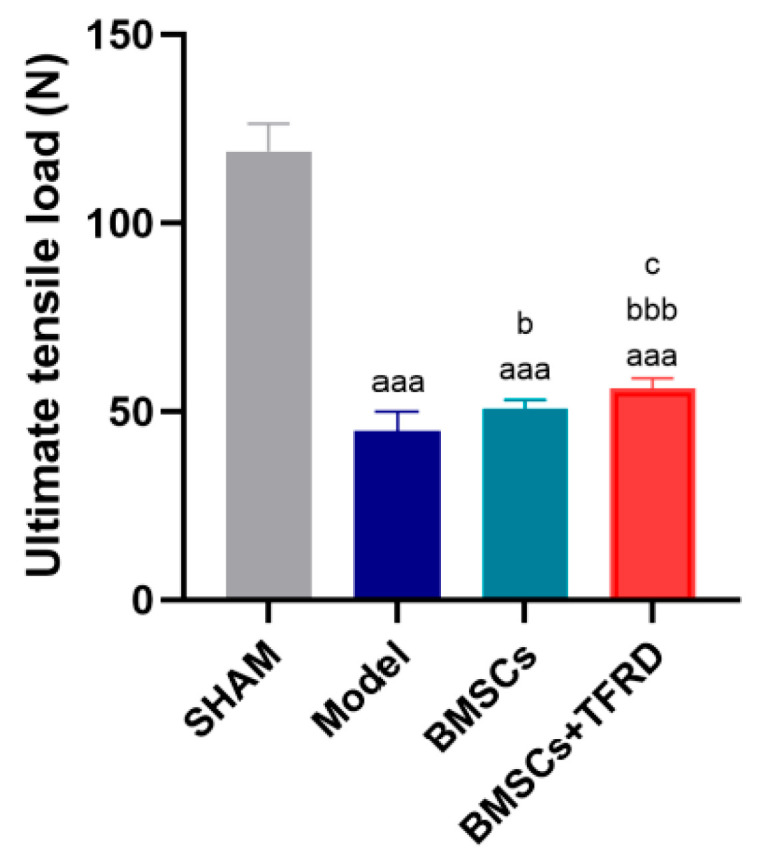
Comparison of tensile strength at the Achilles tendon–calcaneus interface among different experimental groups at 8 weeks post-operation. ^aaa^ *p* < 0.001 vs. SHAM group; ^b^ *p* < 0.05, ^bbb^ *p* < 0.001 vs. Model group; ^c^ *p* < 0.05 vs. BMSCs group.

After 8 weeks of intervention, the ultimate tensile load at the Achilles tendon–calcaneus interface was evaluated. As shown in Figure 15, compared to the SHAM group, the Model, BMSCs, and BMSCs + TFRD groups all exhibited significantly reduced ultimate tensile strength (*p* < 0.001). However, when compared to the Model group, the BMSCs group showed a significant increase in tensile strength (*p* < 0.05), and this enhancement was even more pronounced in the BMSCs + TFRD group (*p* < 0.001). Furthermore, the BMSCs + TFRD group exhibited significantly greater tensile strength than the BMSCs group alone (*p* < 0.05).

These findings indicate that both BMSCs and the combined BMSCs + TFRD treatment significantly improved the biomechanical integrity of the tendon–bone interface, with the combinatory approach showing superior mechanical restoration over BMSC monotherapy.

## 4. Discussion

This study systematically investigated the potential of TFRD in combination with BMSCs to promote TBI regeneration through both in vitro and in vivo experiments. Owing to their multipotent differentiation capacity into osteoblasts, chondrocytes, and fibroblasts [29,30], BMSCs are considered ideal seed cells for TBI regeneration. Previous studies have shown that TFRD can enhance the biological activity of BMSCs through multiple mechanisms, thus supporting their reparative effects in tendon–bone healing [31]. Our in vitro findings confirmed that TFRD significantly promotes BMSC proliferation, migration, and lineage differentiation. In addition, TFRD stimulated HUVEC proliferation and tube formation, indicating its potential to improve the regenerative microenvironment.

Given that in vitro models fail to replicate the complex mechanical loading, cell–matrix interactions, and inflammatory milieu of in vivo TBI, we further established a rat tendon–bone injury model to validate the effects of TFRD + BMSCs in a physiologically relevant context. This model faithfully recapitulates the pathological features of clinical TBI, including the progression from injury to fibrocartilage regeneration [25,32], providing a robust platform for mechanistic investigation.

Cell proliferation provides the foundation for subsequent migration, differentiation, and matrix remodeling [33]. In our study, 5 mg/L TFRD consistently enhanced BMSC viability across multiple time points (12 h, 24 h, 48 h), indicating this dose as optimal for downstream applications. BMSC migration is critical for their homing to injury sites [34]; our scratch assays demonstrated that TFRD significantly promoted BMSC motility, suggesting a supportive role in directing cellular recruitment.

The key to tendon–bone healing is the restoration of the normal structure and function of the TBI, in which bone tissue regeneration is a crucial component [35]. The results of our study demonstrate that TFRD effectively promotes the osteogenic differentiation of BMSCs, thereby providing a vital cellular basis for tendon–bone healing and contributing to the acceleration of injury repair and functional recovery. Previous studies have indicated that TFRD synergistically regulates the osteogenic potential of BMSCs through multiple key signaling pathways, primarily involving modulation at the level of Wnt/β-catenin, RunX2, PPARγ, and miRNAs [36]. Specifically, TFRD can activate the canonical Wnt signaling pathway by upregulating the expression of β-catenin and RunX2 to promote BMSC osteogenic differentiation, while concurrently downregulating the expression of PPARγ to suppress the adipogenic tendency and enhance bone formation capacity [37]. Furthermore, Shen et al. discovered that TFRD can also downregulate the expression of *miR-93-5p*, which in turn relieves the inhibition of Frizzled-6 (FZD6), further activating the Wnt signaling pathway and ultimately promoting the osteogenic differentiation of BMSCs [38]. These findings suggest that in the process of enhancing the osteogenic differentiation capacity of BMSCs, TFRD likely acts through a coordinated, multi-level, and multi-target mechanism to holistically promote bone tissue reconstruction at the TBI site.

The TBI possesses a characteristic multi-layered structure, where the fibrocartilage layer serves as a critical transitional buffer connecting the tendon and bone. The regeneration of this layer is essential for improving interfacial biomechanical properties and enhancing the stability of the tendon–bone junction [39]. BMSCs have a strong chondrogenic differentiation potential and can promote the formation of this fibrocartilage layer, thereby improving the biomechanical performance of the TBI [34]. In our chondrogenic differentiation experiments, we found that TFRD intervention significantly enhanced the formation of cartilaginous matrix by BMSCs, further indicating that TFRD not only promotes osteogenic differentiation but also plays a positive role in inducing the chondrogenic phenotype of BMSCs. Although the specific mechanisms by which TFRD promotes the chondrogenic differentiation of BMSCs are not yet well-understood, we speculate that based on its established roles in cartilage repair, anti-inflammation, and anti-oxidation, it may indirectly facilitate BMSC chondrogenesis by modulating SOX-9 expression, improving the local microenvironment, and suppressing inflammatory responses. In our future research, we will conduct in-depth investigations to validate these potential molecular mechanisms and signaling pathways.

Angiogenesis is equally critical for supplying oxygen, nutrients, and signaling factors [35]. We demonstrated that TFRD promoted HUVEC proliferation, migration, and tube formation. These results align with prior findings that TFRD enhances VEGFA expression, thereby promoting neovascularization [26]. Although VEGFA expression was not directly measured in this study, based on our observation that TFRD promotes HUVEC viability and tube formation, and consistent with previous research, we speculate that TFRD may enhance the angiogenic capacity of endothelial cells in the short term by upregulating pro-angiogenic factors such as VEGFA.

Histological staining further demonstrated that TFRD + BMSCs treatment resulted in better-aligned collagen fibers, enhanced fibrocartilage formation, and improved matrix deposition. These observations support previous studies that showed optimized TBI architecture contributes to enhanced biomechanical integrity [40,41,42].

Immunohistochemistry confirmed that TFRD + BMSCs significantly upregulated the expression of COL I, COL II, and SOX-9, which are key markers of tendon, cartilage, and chondrocyte function, respectively [43,44]. These findings suggest that TFRD promotes both fibrous and cartilaginous matrix remodeling, thus enhancing structural stability and functional recovery at the TBI.

VEGFA expression analysis revealed a dynamic regulation during healing. Although upregulated in early stages to promote vascularization, its expression in the BMSCs + TFRD group was reduced at later stages, suggesting a shift toward vascular remodeling and tissue maturation [45,46]. This adaptive regulation likely contributes to a stable microenvironment.

Serum cytokine profiling showed that BMSCs + TFRD significantly suppressed pro-inflammatory IL-6 and upregulated anti-inflammatory and pro-regenerative cytokines IL-10 and TGF-β. These factors are essential for creating a supportive microenvironment for stem cell survival, differentiation, and matrix synthesis [45,47,48,49,50]. The results further validate the immunomodulatory and regenerative potential of this combined therapy. Cytokine dynamics and readout sensitivity. The modest differences observed in serum IL-6, IL-10, and TGF-β at the endpoint are consistent with the phase-dependent nature of tendon/tendon–bone healing, where pro-inflammatory cytokines (e.g., IL-6) typically rise early and decline during repair/remodeling, while regulatory factors (e.g., IL-10, TGF-β) predominate later to facilitate matrix remodeling and resolution. Moreover, serum readouts are generally less sensitive than local interface histology/IHC for detecting immune modulation in Achilles tendon–bone models; thus, pronounced local improvements can coexist with subtle systemic changes at late time points. These considerations align with recent reviews and experimental studies on tendon/tendon–bone healing [51].

Tensile testing of the tendon–bone junction is a pivotal method for assessing the quality of tendon–bone healing [52]. The ultimate failure load provides a direct measure of the final level of functional recovery after tendon–bone interface (TBI) repair. This functional restoration is a direct manifestation of an optimized histological structure and an improved microenvironment. Previous research on the biomechanical properties and healing strength of the tendon–bone junction has provided a valuable context for our study. For instance, a study by Li et al. reported that in a rat model of tendon–bone healing, the application of BMSCs alone significantly enhanced the ultimate failure load of the junction at 4 and 8 weeks post-surgery. This finding highlights the crucial role of BMSCs in promoting tendon–bone healing and also informed our selection of time points for biomechanical assessment [53]. Our results demonstrated that both the BMSC group and the BMSCs + TFRD group significantly improved the biomechanical properties of the Achilles tendon-calcaneus junction. Compared to BMSC monotherapy, the combined therapy of TFRD and BMSCs exhibited a more significant effect in enhancing the ultimate failure load. This result indicates a synergistic effect between TFRD and BMSCs in promoting tendon–bone healing, leading to a more effective restoration of the TBI’s mechanical performance. Furthermore, Han et al., using a mouse model of anterior cruciate ligament reconstruction, investigated the effects of TFRD and found that it promoted the osteogenic differentiation of BMSCs via the ERRα/β-Gga1-TGF-β/MAPK pathway, thereby significantly increasing the tensile strength of the tendon–bone junction [31].

In summary, this study systematically validated the synergistic effect of TFRD combined with BMSCs in TBI repair. Our in vitro experiments demonstrated that TFRD significantly promotes the proliferation, migration, and both osteogenic and chondrogenic differentiation of BMSCs, while also enhancing the angiogenic capacity of HUVECs. This provides a cellular basis and vascular support for the multi-tissue reconstruction of the TBI. In vivo studies further confirmed that the combined TFRD + BMSCs intervention effectively enhances the structural remodeling of the TBI region, including the orderly arrangement of collagen fibers, the formation of fibrocartilage, and the process of matrix mineralization, ultimately leading to a significant improvement in the biomechanical properties of the tendon–bone junction. Mechanistically, this intervention promotes angiogenesis through the dynamic regulation of VEGFA expression, thereby improving local oxygen and nutrient supply. Concurrently, it fosters a pro-regenerative microenvironment by downregulating the pro-inflammatory cytokine IL-6 and upregulating the anti-inflammatory and pro-reparative factors IL-10 and TGF-β. These findings further substantiate the synergistic and enhancing effects of the combined application of TFRD and BMSCs in tendon–bone healing, providing important experimental evidence for optimizing intervention strategies for tendon–bone injuries.

Despite these encouraging results, several limitations remain. First, TFRD is a complex mixture of flavonoid monomers, and the specific active components responsible for the observed effects are yet to be identified. Future studies should aim to isolate and characterize individual compounds and elucidate their molecular targets and pathways. Second, while we focused on VEGFA, IL-6, IL-10, and TGF-β, other regulatory molecules such as BMPs, FGFs, TNF-α, MMPs, and chemokines should also be included in future investigations to build a more comprehensive regulatory network.

## 5. Conclusions

The in vitro results indicate that TFRD can enhance the proliferation, migration, and osteogenic and chondrogenic differentiation of BMSCs, while also promoting the angiogenic functions of HUVECs, suggesting its role in providing the cellular basis and vascular support required for repair. The in vivo experiments further validated that the combination of TFRD and BMSCs effectively promotes collagen synthesis and cartilage matrix deposition, improves tissue structural reconstruction, optimizes the matrix microenvironment and cytokine levels, increases the mechanical strength of the tendon–bone junction, and significantly enhances the repair capacity of the TBI.

## Data Availability

All data relevant to the study are included in the article. In addition, the datasets used and/or analyzed during the current study are available from the corresponding author on reasonable request.

## Supplementary Materials

The following supporting information can be downloaded at: https://www.mdpi.com/article/10.3390/biology14111593/s1, Figure S1: Male Sprague-Dawley (SD) rat; Figures S2 and S3: Shaving and sterilizing the surgical site.

## References

[1] Yang C., Teng Y., Geng B., Xiao H., Chen C., Chen R., Yang F., Xia Y. (2023). Strategies for promoting tendon-bone healing: Current status and prospects. Front. Bioeng. Biotechnol..

[2] Feltri P., Monteleone A.S., Audige L., Marbach F., Filardo G., Candrian C. (2024). Patients with rotator cuff tears present a psychological impairment, not only a functional deficit: A systematic review. Int. Orthop..

[3] Lei T., Zhang T., Ju W., Chen X., Heng B.C., Shen W., Yin Z. (2021). Biomimetic strategies for tendon/ligament-to-bone interface regeneration. Bioact. Mater..

[4] Dang G.P., Qin W., Wan Q.Q., Gu J.T., Wang K.Y., Mu Z., Gao B., Jiao K., Tay F.R., Niu L.N. (2023). Regulation and Reconstruction of Cell Phenotype Gradients Along the Tendon-Bone Interface. Adv. Funct. Mater..

[5] Jiang F., Zhao H., Zhang P., Bi Y., Zhang H., Sun S., Yao Y., Zhu X., Yang F., Liu Y. (2024). Challenges in tendon–bone healing: Emphasizing inflammatory modulation mechanisms and treatment. Front. Endocrinol..

[6] Figueroa D., Figueroa F., Calvo R., Vaisman A., Ahumada X., Arellano S. (2015). Platelet-rich plasma use in anterior cruciate ligament surgery: Systematic review of the literature. Arthrosc. J. Arthrosc. Relat. Surg..

[7] Xu J., Ye Z., Han K., Zheng T., Zhang T., Dong S., Jiang J., Yan X., Cai J., Zhao J. (2022). Infrapatellar fat pad mesenchymal stromal cell–derived exosomes accelerate tendon-bone healing and intra-articular graft remodeling after Anterior Cruciate Ligament Reconstruction. Am. J. Sports Med..

[8] Yu C., Sun L., Gao H., Sheng H., Feng X., Yang X., Li J., Kong Q., Hao Y., Feng S. (2024). Rotator cuff repair with all-suture anchor enhances biomechanical properties and tendon-bone integration in a rabbit model. Heliyon.

[9] Chen G., Zheng Q., Liu M., He H., Ju X., Jiang K. (2023). Effect of Kartogenin combined with adipose-derived stem cells on tendon-bone healing after anterior cruciate ligament reconstruction. Chin. J. Reparative Reconstr. Surg..

[10] Wang X., Wu Y., Zhang X., Shi Z., Yang T., Xiong B., Lu X., Zhao D. (2024). Expression and action mechanism of stromal cell-derived factor 1 in tendon-bone healing of rabbit rotator cuff. Chin. J. Tissue Eng. Res..

[11] Han L., Fu H., Li P., Hu Y., Liu J.J. (2021). Biological mechanism of extracorporeal shock wave combined with platelet-rich plasma on the healing of reconstructed tendon bone in a rabbit model of rotator cuff tear. INDIAN J. Pharm. Sci..

[12] Huang Y., He B., Wang L., Yuan B., Shu H., Zhang F., Sun L. (2020). Bone marrow mesenchymal stem cell-derived exosomes promote rotator cuff tendon-bone healing by promoting angiogenesis and regulating M1 macrophages in rats. Stem Cell Res. Ther..

[13] Zhu Y., Yan J., Zhang H., Cui G. (2023). Bone marrow mesenchymal stem cell-derived exosomes: A novel therapeutic agent for tendon-bone healing. Int. J. Mol. Med..

[14] Xu Y., Zhang W.-X., Wang L.-N., Ming Y.-Q., Li Y.-L., Ni G.-X. (2021). Stem cell therapies in tendon-bone healing. World J. Stem Cells.

[15] Chow S.K.-H., Gao Q., Pius A., Morita M., Ergul Y., Murayama M., Shinohara I., Cekuc M.S., Ma C., Susuki Y. (2024). The advantages and shortcomings of stem cell therapy for enhanced bone healing. Tissue Eng. Part C Methods.

[16] Zhang T., Yan S., Song Y., Chen C., Xu D., Lu B., Xu Y. (2022). Exosomes secreted by hypoxia-stimulated bone-marrow mesenchymal stem cells promote grafted tendon-bone tunnel healing in rat anterior cruciate ligament reconstruction model. J. Orthop. Transl..

[17] Zhao Y., Cai X., Sun J., Bi W., Yu Y. (2024). Active components and mechanisms of total flavonoids from *Rhizoma drynariae* in enhancing cranial bone regeneration: An investigation employing serum pharmacochemistry and network pharmacology approaches. J. Ethnopharmacol..

[18] Mou M., Li C., Huang M., Zhao Z., Ling H., Zhang X., Wang F., Yin X., Ma Y. (2015). Antiosteoporotic effect of the Rhizome of Drynaria Fortunei (Kunze)(Polypodiaceae) With Special emphasis on its modes of action. Acta Pol. Pharm..

[19] Guo J., Liang Y., Fan H., Huang H., Liu J., Peng C., Shu J. (2025). A comprehensive review of Drynariae rhizoma: Botany, traditional applications, and active flavonoid components. J. Pharm. Pharmacol..

[20] Zhang Y., Lei X., Xu H., Liu G., Wang Y., Sun H., Geng F., Zhang N. (2022). Tissue Distribution of Total Flavonoids Extracts of Drynariae Rhizoma in Young and Old Rats by UPLC–MS/MS Determination. J. Anal. Methods Chem..

[21] Wang J., Li J., Liu G. (2021). Mechanism of total flavonoids of *Rhizoma drynariae* in multiplication response and differentiation of BMSCs in rats. Acad. J. Chin. PLA Med. Sch..

[22] Long Y.-l., Tian Q.-h. (2022). Effects of total flavonoids of Drynariae Rhizoma on osteogenic differentiation potential of canine bone marrow mesenchymal stem cells in hypoxic environment. Acta Vet. Zootech. Sin..

[23] Lv W., Yu M., Yang Q., Kong P., Yan B. (2021). Total flavonoids of *Rhizoma drynariae* ameliorate steroid-induced avascular necrosis of the femoral head via the PI3K/AKT pathway. Mol. Med. Rep..

[24] Zhou Q., Zeng X., Huang D., Li J., Tang M., Li S. (2021). Research progress on the chemical composition and biological activity of Drynariae Rhizoma. World Sci. Technol. Mod. Tradit. Chin. Med..

[25] Xu B., Wang Y., He G., Tang K.-l., Guo L., Chen W. (2024). A novel and efficient murine model for investigating tendon-to-bone healing. J. Orthop. Surg. Res..

[26] Shen Z., Chen Z., Li Z., Zhang Y., Jiang T., Lin H., Huang M., Chen H., Feng J., Jiang Z. (2020). Total flavonoids of *Rhizoma drynariae* enhances angiogenic-osteogenic coupling during distraction osteogenesis by promoting type H vessel formation through PDGF-BB/PDGFR-β instead of HIF-1α/VEGF Axis. Front. Pharmacol..

[27] Ide J., Kikukawa K., Hirose J., Iyama K.-i., Sakamoto H., Fujimoto T., Mizuta H. (2009). The effect of a local application of fibroblast growth factor-2 on tendon-to-bone remodeling in rats with acute injury and repair of the supraspinatus tendon. J. Shoulder Elb. Surg..

[28] Dominici M., Le Blanc K., Mueller I., Slaper-Cortenbach I., Marini F., Krause D., Deans R., Keating A., Prockop D., Horwitz E. (2006). Minimal criteria for defining multipotent mesenchymal stromal cells. The International Society for Cellular Therapy position statement. Cytotherapy.

[29] Wang L., Guan C., Zhang T., Zhou Y., Liu Y., Hu J., Xu D., Lu H. (2024). Comparative effect of skeletal stem cells versus bone marrow mesenchymal stem cells on rotator cuff tendon-bone healing. J. Orthop. Transl..

[30] Shen S., Lin Y., Sun J., Liu Y., Chen Y., Lu J. (2024). A New Tissue Engineering Strategy to Promote Tendon–bone Healing: Regulation of Osteogenic and Chondrogenic Differentiation of Tendon-derived Stem Cells. Orthop. Surg..

[31] Han L., Wang C., Wang T., Hu Y., Wang H. (2024). Total flavonoids of *Rhizoma drynariae* improves tendon-bone healing for anterior cruciate ligament reconstruction in mice and promotes the osteogenic differentiation of bone mesenchymal stem cells by the ERR1/2-Gga1-TGF-β/MAPK pathway. Environ. Toxicol..

[32] Kovacevic D., Fox A.J., Bedi A., Ying L., Deng X.-H., Warren R.F., Rodeo S.A. (2011). Calcium-phosphate matrix with or without TGF-β3 improves tendon-bone healing after rotator cuff repair. Am. J. Sports Med..

[33] Song F., Jiang D., Wang T., Wang Y., Chen F., Xu G., Kang Y., Zhang Y. (2017). Mechanical loading improves tendon-bone healing in a rabbit anterior cruciate ligament reconstruction model by promoting proliferation and matrix formation of mesenchymal stem cells and tendon cells. Cell. Physiol. Biochem..

[34] Zhang C., Jiang C., Jin J., Lei P., Cai Y., Wang Y. (2023). Cartilage fragments combined with BMSCs-Derived exosomes can promote tendon-bone healing after ACL reconstruction. Mater. Today Bio.

[35] Chen Z., Jin M., He H., Dong J., Li J., Nie J., Wang Z., Xu J., Wu F. (2023). Mesenchymal stem cells and macrophages and their interactions in tendon-bone healing. J. Orthop. Transl..

[36] Li S., Zhou H., Hu C., Yang J., Ye J., Zhou Y., Li Z., Chen L., Zhou Q. (2021). Total flavonoids of *Rhizoma drynariae* promotes differentiation of osteoblasts and growth of bone graft in induced membrane partly by activating wnt/β-catenin signaling pathway. Front. Pharmacol..

[37] Su H., Liu L., Yan Z., Guo W., Huang G., Zhuang R., Pan Y. (2025). Therapeutic potential of total flavonoids of Rhizoma Drynariae: Inhibiting adipogenesis and promoting osteogenesis via MAPK/HIF-1α pathway in primary osteoporosis. J. Orthop. Surg. Res..

[38] Shen X.-J., Li L.-L., Lu Y., Wang L.-W., Yuan R.-T., Guo Q.-Y., Zhao P. (2023). The role and mechanism of miR-93-5p in osteogenic differentiation of rabbit bone marrow mesenchymal stem cells mediated by total flavonoids of rhizoma drynariae. China J. Oral Maxillofac. Surg..

[39] Chen K., Liu Z., Zhou X., Zheng W., Cao H., Yang Z., Wang Z., Ning C., Li Q., Zhao H. (2025). Hierarchy Reproduction: Multiphasic Strategies for Tendon/Ligament–Bone Junction Repair. Biomater. Res..

[40] Kanazawa T., Gotoh M., Ohta K., Honda H., Ohzono H., Shimokobe H., Shiba N., Nakamura K.-i. (2016). Histomorphometric and ultrastructural analysis of the tendon-bone interface after rotator cuff repair in a rat model. Sci. Rep..

[41] Xu B., Wang Y., He G., Tao X., Gao S., Zhou M., Tang Y., Tang K.-l., Guo L., Chen W. (2025). An Aligned-to-Random PLGA/Col1-PLGA/nHA Bilayer Electrospun Nanofiber Membrane Enhances Tendon-to-Bone Healing in a Murine Model. Am. J. Sports Med..

[42] Teng C., Zhou C., Xu D., Bi F. (2016). Combination of platelet-rich plasma and bone marrow mesenchymal stem cells enhances tendon–bone healing in a rabbit model of anterior cruciate ligament reconstruction. J. Orthop. Surg. Res..

[43] Watanabe G., Yamamoto M., Taniguchi S., Sugiyama Y., Hirouchi H., Ishizuka S., Kitamura K., Mizoguchi T., Takayama T., Hayashi K. (2023). Chronological changes in the expression and localization of Sox9 between achilles tendon injury and functional recovery in mice. Int. J. Mol. Sci..

[44] Zou J., Yang W., Cui W., Li C., Ma C., Ji X., Hong J., Qu Z., Chen J., Liu A. (2023). Therapeutic potential and mechanisms of mesenchymal stem cell-derived exosomes as bioactive materials in tendon–bone healing. J. Nanobiotechnol..

[45] Xu Q., Sun W.-X., Zhang Z.-F. (2019). High expression of VEGFA in MSCs promotes tendon-bone healing of rotator cuff tear via microRNA-205-5p. Eur. Rev. Med. Pharmacol. Sci..

[46] Liu X., Zhu B., Li Y., Liu X., Guo S., Wang C., Li S., Wang D. (2021). The role of vascular endothelial growth factor in tendon healing. Front. Physiol..

[47] Gao H., Wang L., Jin H., Lin Z., Li Z., Kang Y., Lyu Y., Dong W., Liu Y., Shi D. (2022). Regulating macrophages through immunomodulatory biomaterials is a promising strategy for promoting tendon-bone healing. J. Funct. Biomater..

[48] Zou M., Wang J., Shao Z. (2023). Therapeutic potential of exosomes in tendon and tendon–bone healing: A systematic review of preclinical studies. J. Funct. Biomater..

[49] Chen Q.-Q., Yan L., Wang C.-Z., Wang W.-H., Shi H., Su B.-B., Zeng Q.-H., Du H.-T., Wan J. (2013). Mesenchymal stem cells alleviate TNBS-induced colitis by modulating inflammatory and autoimmune responses. World J. Gastroenterol..

[50] Zhao K., Chen M., Liu T., Zhang P., Wang S., Liu X., Wang Q., Sheng J. (2021). *Rhizoma drynariae* total flavonoids inhibit the inflammatory response and matrix degeneration via MAPK pathway in a rat degenerative cervical intervertebral disc model. Biomed. Pharmacother..

[51] Darrieutort-Laffite C., Blanchard F., Soslowsky L.J., Le Goff B. (2024). Biology and physiology of tendon healing. Jt. Bone Spine.

[52] Hettrich C.M., Gasinu S., Beamer B.S., Stasiak M., Fox A., Birmingham P., Ying O., Deng X.-H., Rodeo S.A. (2014). The effect of mechanical load on tendon-to-bone healing in a rat model. Am. J. Sports Med..

[53] Li Y.-G., Wei J.-N., Lu J., Wu X.-T., Teng G.-J. (2011). Labeling and tracing of bone marrow mesenchymal stem cells for tendon-to-bone tunnel healing. Knee Surg. Sports Traumatol. Arthrosc..

