# Supplementary material for: Total Flavonoids of Rhizoma drynariae Enhance Bone Marrow Mesenchymal Stem Cell-Mediated Tendon–Bone Healing by Promoting Tissue Regeneration, Angiogenesis, and Modulation of Cytokine Expression
Source: Biology (Basel). 2025 Nov 14;14(11):1593. doi: 10.3390/biology14111593 (PMC12649899; doi:10.3390/biology14111593)
Supplement: Supplementary file 1 [file biology-14-01593-s001.zip › biology-3861343-supplementary.pdf]

S1 **S2** **S3**

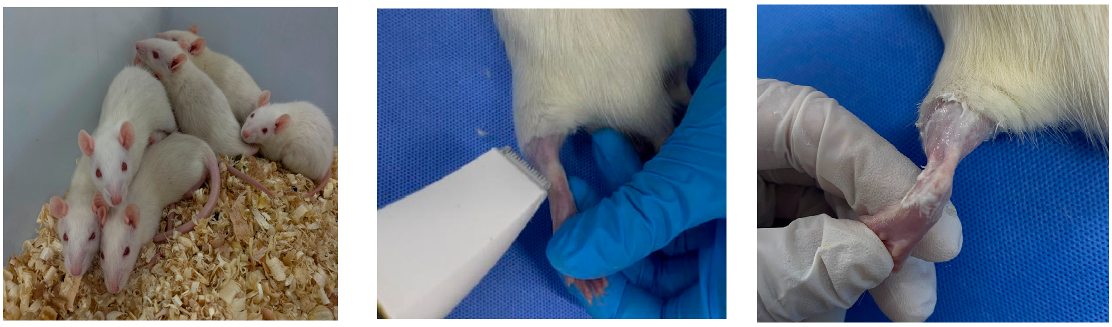

Figure S1: Male Sprague-Dawley (SD) rat; Figures S2 and S3: Shaving and sterilizing the surgical site
